# Pattern, clinical features and response to corticoids of glomerular diseases in a Brazilian population. An analytical cross-sectional study: Perfil, características clínicas e resposta a corticoides das doenças glomerulares em uma população brasileira. Um estudo transversal analítico

**DOI:** 10.1590/1516-3180.2013.7360006

**Published:** 2014-11-28

**Authors:** Anaiara Lucena Queiroz, Dulce Maria Sousa Barreto, Geraldo Bezerra da Silva, José Edísio da Silva Tavares, Francisco Israel Costa, Régia Maria do Socorro Vidal Patrocínio, Elizabeth De Francesco Daher, Paulo Roberto Carvalho de Almeida

**Affiliations:** I MD, Postgraduate Pathology Program, Universidade Federal do Ceará (UFCE), Fortaleza, Ceará, Brazil.; II MD. Nephrologist and Attending Physician, Division of Nephrology, Hospital Geral de Fortaleza (HGF), Fortaleza, Ceará, Brazil.; III MD, PhD. Adjunct Professor in the Medicine Course and Post-Graduation Program in Collective Health, Universidade de Fortaleza (UNIFOR), Fortaleza, Ceará, Brazil.; IV MD. Attending Physician, Department of Internal Medicine, Universidade Federal do Ceará (UFCE), Fortaleza, Ceará, Brazil.; V MD. Pathologist in the Postgraduate Pathology Program, Universidade Federal do Ceará (UFCE), Fortaleza, Ceará, Brazil.; VI MD, PhD. Adjunct Professor, Department of Internal Medicine, Universidade Federal do Ceará (UFCE), Fortaleza, Ceará, Brazil.; VII MD, PhD. Adjunct Professor in the Postgraduate Pathology Program, Universidade Federal do Ceará (UFCE), Fortaleza, Ceará, Brazil.

**Keywords:** Glomerulonephritis, Proteinuria, Renal insufficiency, Therapeutics, Glomerular filtration rate

## Abstract

**CONTEXT AND OBJECTIVE:**

Glomerular disease registries are increasing all around the world. The aim of this study was to evaluate the clinical characteristics and treatment response among patients with glomerular diseases followed up in a tertiary hospital in Brazil.

**DESIGN AND SETTING:**

Analytical cross-sectional study; tertiary-level public hospital.

**METHODS:**

This study included patients with glomerular diseases followed up at a tertiary hospital in Fortaleza, northeastern Brazil. Clinical and laboratory data on each patient were registered. The response to specific treatment was evaluated after 3, 6 and 12 months.

**RESULTS:**

The study included 168 patients of mean age 37 ± 14 years. The most prevalent glomerular diseases were focal segmental glomerulosclerosis [FSGS] (19.6%), minimal change disease [MCD] (17.9%), membranous nephropathy [MN] (16.7%) and lupus nephritis [LN] (11.9%). The main clinical presentations were nephrotic proteinuria (67.3%) and renal insufficiency (17.9%). The mean proteinuria value decreased after the treatment began. Regarding 24-hour proteinuria on admission, there was no significant difference between patients with a good response and those with no response (7,448 ± 5,056 versus 6,448 ± 4,251 mg/24 h, P = 0.29). The glomerular disease with the highest remission rate was MCD (92%). Absence of interstitial fibrosis presented a strong correlation with remission (remission in patients without fibrosis = 83.4% versus 16.3% in those with fibrosis, P = 0.001).

**CONCLUSIONS:**

The present study found that the most frequent glomerular disease was FSGS, followed by MCD, MN and LN. The presence of interstitial fibrosis was a predictor of poor therapeutic response.

## RESUMO

**CONTEXTO E OBJETIVO:**

Registros de glomerulopatias estão aumentando em todo o mundo. O objetivo deste estudo é avaliar as características clínicas e a resposta do tratamento de pacientes com glomerulopatias acompanhados em um hospital terciário no Brasil.

**TIPO DE ESTUDO E LOCAL:**

Estudo transversal analítico. Hospital público terciário.

**MÉTODOS:**

O estudo incluiu pacientes com glomerulopatias acompanhados em um hospital terciário de Fortaleza, Ceará, Brasil. Foi realizado registro dos dados clínicos e laboratoriais para cada paciente. A resposta ao tratamento específico foi avaliada após 3, 6 e 12 meses.

**RESULTADOS:**

Foram incluídos 168 pacientes, com média de idade de 37 ± 14 anos. A glomerulopatia mais prevalente foi a glomerulosclerose segmentar e focal [GESF] (19,6%), seguida pela doença de lesão mínima [DLM] (17,9%), nefropatia membranosa [NM] (16,7%) e nefrite lúpica [NL] (11,9%). As principais manifestações clínicas foram proteinúria nefrótica (67,3%) e insuficiência renal (17,9%). A média dos valores de proteinúria diminuiu após o início do tratamento. Com relação à proteinúria de 24 horas na admissão, não houve diferença significativa entre os pacientes com boa resposta ao tratamento e aqueles sem resposta (7.448 ± 5.056 *versus* 6.448 ± 4.251 mg/24 h, P = 0,29). A doença com maior índice de remissão foi a DLM (92%). A ausência de fibrose intersticial apresentou forte correlação com a remissão (remissão em pacientes sem fibrose = 83,4% *versus* 16,3% naqueles com fibrose, P = 0,001).

**CONCLUSÕES:**

O presente estudo encontrou como glomerulopatia mais frequente a GESF, seguida da DLM, NM e NL. A presença de fibrose intersticial foi um preditor de pobre resposta terapêutica.

## INTRODUCTION

Glomerular diseases are still one of the main causes of chronic kidney diseases in many parts of the world. North American and Brazilian data show that glomerular diseases are currently the third commonest end-stage renal disease, after diabetes and hypertension.^1^^,^^2^ Glomerular diseases registries are increasing all around the world, including in Brazil.^3^^,^^4^^,^^5^^,^^6^

The variability in glomerular disease prevalence in different regions of the world can be correlated with indications for renal biopsy. In Spain, higher prevalence of immunoglobulin A (IgA) nephropathy was observed among patients with minimal urinary changes, while membranous nephropathy was more frequent among adult patients with nephrotic syndrome.^3^

The distribution of glomerular diseases associated with nephrotic syndrome in adults in Brazil has changed over the last few years. Studies from the 1970s demonstrated that membranoproliferative glomerulonephritis was the most prevalent type because of the high prevalence of schistosomiasis and other parasitic diseases.^7^^,^^8^^,^^9^ More recent studies have shown an increase in the incidence of focal and segmental glomerulosclerosis, and this is currently the most prevalent form of glomerulonephritis in Brazil and other regions of the world.^10^^,^^11^^,^^12^ So far, there have been few studies on the distribution of glomerular diseases in the northeastern region of Brazil.

## OBJECTIVE

The aim of this study was to evaluate the clinical pattern and treatment response among patients with biopsy-proven glomerular diseases who were followed up at a tertiary hospital in Fortaleza, Ceará, northeastern Brazil.

## METHODS

### Study population

The study population included all patients with a biopsy-proven diagnosis of glomerular disease who were followed up at the nephrology outpatient clinic of the General Hospital of Fortaleza, northeastern Brazil, between February 2010 and September 2011. This was an analytical cross-sectional study. Data were collected during routine medical visits.

### Clinical and laboratory evaluation

A record of clinical and laboratory data was made for each patient. The clinical data included name, age, gender, beginning of follow-up, presence of nephrotic syndrome, renal insufficiency, type of treatment and renal biopsy results. Laboratory tests were performed on admission and 3, 6 and 12 months after biopsy, including serum creatinine, 24-hour proteinuria, serum proteins and cholesterol. We also evaluated the prognostic factors of refractoriness and response to corticoid therapy.

### Definitions

Nephrotic syndrome was defined as the presence of 24-hour proteinuria > 3.5 g, serum albumin < 3.5 g/dl and hypercholesterolemia (total cholesterol > 200 mg/dl), in association with clinically evident edema.^13^

The glomerular disease was classified as primary at the time of diagnosis if the patient did not present any systemic disease, was serologically negative for hepatitis B and C viruses and for HIV, was negative for antinuclear antibodies and did not have any family history of hematuria.^14^ The presence of primary neoplasia was ruled out by performing abdominal ultrasound, mammography, digestive endoscopy, colonoscopy and prostate-specific antigen assaying.

Renal excretory function was determined based on serum creatinine. The glomerular filtration rate was estimated through the MDRD (Modified Diet in Renal Disease) formula: 186 X (Cr) - 1.154 X (age) - 0.203 X (0.742, if female) X (1.210, if African-American).^15^ Renal insufficiency on admission was defined as a glomerular filtration rate < 60 ml/min/1.73 m^2^.^16^

The renal function response after treatment end was considered to be favorable if there was a 15% decrease in serum creatinine or stabilization of creatinine in patients who had presented increasing levels of creatinine before the specific treatment.^17^

The response to specific treatment was classified in accordance with the following definitions, when the patient had undergone at least six months of treatment:^17^

- Complete remission was defined as a proteinuria decrease to less than 0.3 g/day and a maximum variation in serum creatinine of 10% in relation to its initial level;
- Partial remission was defined as a decrease of at least 50% in the initial proteinuria level and proteinuria < 3.0 g/day and a maximum variation in serum creatinine of 10% in relation to its initial level;
- Resistance to treatment was defined as a proteinuria decrease < 50% or any decrease associated with proteinuria > 3 g/24 h, together with a serum creatinine variation of no more than 10% in relation to its initial level;
- Deterioration was defined as a proteinuria increase > 50% from its baseline level or an increase in serum creatinine > 10% in relation to its baseline level;
- The response was considered to be undefined when it was not possible to evaluate the patient’s response because of a short period of treatment (less than six months) or when the patient was lost from the follow-up.

### Clinical and laboratory follow-up

Clinical and laboratory follow-ups were performed in the first, third and sixth months after the treatment began, and subsequently at variable intervals, depending on each patient’s characteristics.

### Statistical analysis

The statistical analysis was done using the SPSS software, version 19.0. Central trend and dispersion measurements (means and standard deviations), tables and graphs were used to characterize the parameters studied. Continuous variables were tested for normal distribution by means of the Kolmogorov-Smirnov test. Variables with normal distribution were analyzed using parametric tests (Student’s t test or paired t test), when appropriate. Categorical variables were analyzed using the chi-square test. Variance analysis was done through ANOVA (analysis of variance) test. The significance level was taken to be 5% for two-tailed tests.

### Ethical issues

The present study was reviewed and approved by the institution’s Ethics Committee (protocol 020407/09).

## RESULTS

### Epidemiological characteristics of the study population

Out of a total of 195 patients registered at the nephrology outpatient clinic of the General Hospital of Fortaleza with a confirmed diagnosis of glomerular disease, 168 adhered to the follow-up until the end of the study, of whom 84 (50%) were males. The patients’ mean age was 37 ± 14 years (range 14-77). The patients’ mean ages according to the most prevalent glomerular diseases were 35 ± 15 years (in focal segmental glomerulosclerosis), 32 ± 12 years (minimal change disease), 40 ± 14 years (membranous nephropathy), 34 ± 8 years (lupus nephritis) and 40 ± 9 years (IgA nephropathy) (P = 0.087, one way, ANOVA). The mean length of follow-up was 14.8 ± 12 months (range 1-72 months).

### Prevalence and classification of glomerular diseases

The most prevalent glomerular diseases were: focal segmental glomerulosclerosis (19.6%), minimal change disease (17.9%), membranous nephropathy (16.7%) and lupus nephritis (11.9%). These four glomerular diseases accounted for a total of 111 patients (66.1%).

The less frequent diseases were: IgA nephropathy (7.1%), membranoproliferative glomerulonephritis (6.5%), mesangial glomerulonephritis (6.0%), post-infectious glomerulonephritis (4.8%), crescentic glomerulonephritis (3.6%), amyloidosis (1.8%) and diabetic nephropathy (1.2%). IgM nephropathy, thin basement membrane and HIV-associated nephropathy were found in one patient each (0.6%), as summarized in Table 1.

**Table 1.**
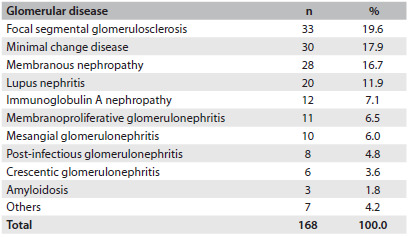
Prevalence of glomerular diseases among patients followed up at the General Hospital of Fortaleza, Brazil

The histopathological patterns of the 168 biopsies revealed higher prevalence of primary glomerular diseases, which corresponded to 124 cases (74.7%), while the secondary diseases comprised 42 cases (25.3%). Among the secondary diseases, lupus nephritis was the most prevalent. The patients with lupus nephritis were classified as class IV (60%), class V (30%) and class III (10%).

Primary glomerular diseases were more frequent among males (69/124 = 55.6%), while secondary diseases were more common among females (29/42 = 69%, P = 0.0057).

### Histopathological findings

The mean number of glomeruli found in the renal biopsies was 20.4 ± 12.5, and 91.7% presented more than 8 glomeruli. The number of arteries ranged from 0 to 8, with a mean of 3 arteries per sample.

Regarding interstitial changes, there was interstitial fibrosis in 77 patients (45.8%), while 41.1% showed no interstitial changes. Among the patients with fibrosis, 62.3% presented mild interstitial fibrosis, 27.2% moderate and 10.3% severe fibrosis.

### Clinical manifestations

The main clinical presentations were nephrotic proteinuria (67.3%), renal insufficiency (17.9%), non-nephrotic proteinuria (6.5%), nephritic syndrome (6%) and persistent hematuria (1.2%), as summarized in Table 2.

**Table 2.**
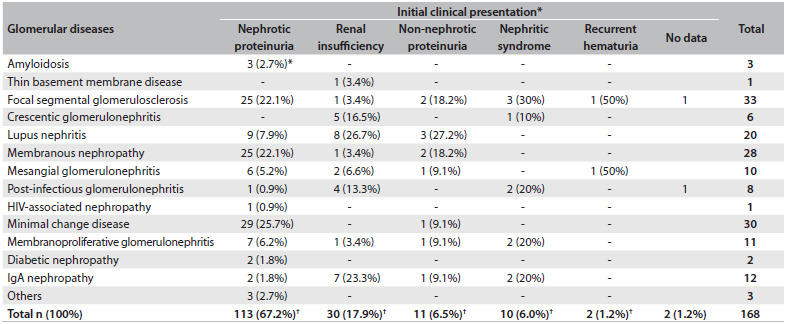
Clinical presentation of glomerular diseases on admission among patients followed up at the General Hospital of Fortaleza, Brazil

*% in relation to the total in the column; ^†^% in relation to the total in general.

Renal insufficiency was the most frequent presentation among patients with crescentic glomerulonephritis (83.3%). Among the patients with IgA nephropathy, 7 (58.3%) had renal insufficiency on admission.

The patients with nephrotic proteinuria had the diagnoses of minimal change disease (17.3%), membranous nephropathy (14.9%) and focal segmental glomerulosclerosis (14.9%).

### Laboratory tests

The mean value for 24-hour proteinuria was 6.5 ± 4.5 g (range: 305-19,500 mg). Membranous nephropathy was the disease with the highest levels of proteinuria (mean: 8,640 mg/24 h). The disease with the lowest level of proteinuria was IgA nephropathy (2,470 mg/24 h). Table 3 shows the means for 24-hour proteinuria in the different histopathological patterns of glomerular diseases.

**Table 3.**
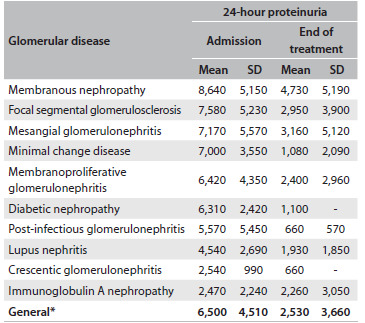
Laboratory values for 24-hour proteinuria on admission and after treatment among patients with glomerular diseases followed up at the General Hospital of Fortaleza, Brazil

^*^Paired t test (P-value < 0.001). SD = standard deviation.

The mean values for proteinuria decreased after the treatment began. The mean proteinuria levels decreased to 4,940 mg/24 h after 3 months of treatment, 2,700 mg/24 h after 6 months and 3,320 mg/24 h after 12 months. Table 4 shows that this decrease was statistically significant for each month, in comparison with the proteinuria baseline levels.

**Table 4.**
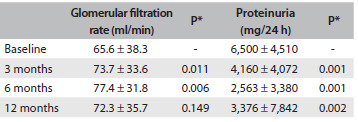
Evaluation of renal function and 24-hour proteinuria on admission and after 3, 6 and 12 months of treatment, among patients with glomerular diseases followed up at the General Hospital of Fortaleza, Brazil

*Paired t test: comparing months 3, 6 and 12 with baseline values.

The mean glomerular filtration rate on admission was 65.6 ml/min. There was an increase to 73.7 ± 33.6 ml/min over the first 3 months and 77.4 ± 31.8 ml/min over the first 6 months. This increase was statistically significant after the third and sixth months, in comparison with the baseline glomerular filtration rate levels (P = 0.011 and P = 0.006, respectively). After 12 months of treatment, the mean glomerular filtration rate was 72.3 ± 35.7, but this increase was not statistically significant (P = 0.149).

Among the patients studied, 77 (45.8%) had some degree of renal insufficiency on admission. The disease with the highest glomerular filtration rate was minimal change disease (89.9 ml/min).

The mean age of patients with renal insufficiency on admission was 40.7 ± 15.2 years, while the mean age of those with a normal glomerular filtration rate was 33.1 ± 13.1 (P = 0.036). The mean proteinuria level among the patients with renal insufficiency on admission was 6,570 ± 4,830 mg/24 h, while the mean proteinuria among those without renal insufficiency was 6,563 ± 4,190 mg/24 h (P = 0.362).

The mean total cholesterol on admission was 316 ± 137 mg/dl (range: 100-1014 mg/dl), and the mean serum albumin was 2.9 ± 0.9 g/dl (range: 1.3-4.7 g/dl). Serological tests for hepatitis B and C viruses and for HIV showed that hepatitis B had been cured in one case and that one case was HIV-positive.

### Treatment

Corticosteroids, including oral prednisone and methylprednisolone, were administered in 70.4% of the cases. Oral prednisone alone, at an initial dose of 1 mg/kg/day, was used in 43.6% of the cases. Antiproteinuric agents were administered to 22.1% of the patients as the sole therapy. Cyclophosphamide in association with corticosteroids was used in 14.7% of the cases. Cyclosporine in association with low-dose corticosteroids was administered in 9.4% of the cases, while mycophenolate mofetil was used in 2.6% of the cases. Table 5 shows the drugs used as the initial treatment.

**Table 5.**
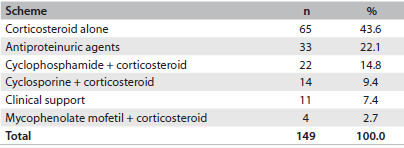
Initial treatment instituted for patients with glomerular diseases followed up at the General Hospital of Fortaleza, Brazil

A second therapeutic approach was necessary in 28.2% of the cases. The immunosuppressants that were most used as rescue therapy were cyclosporine and mycophenolate mofetil (Table 6).

**Table 6.**
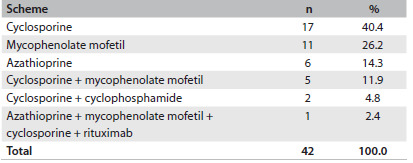
Rescue therapy used for patients with glomerular diseases followed up at the General Hospital of Fortaleza, Brazil

The patients with a good response to therapy were of similar age to those who did not respond (36.23 ± 13.59 versus 36.57 ± 15.21 years; P = 0.910). Regarding 24-hour proteinuria on admission, there was no significant difference between patients with a good response and those with no response (7,448 ± 5,056 versus 6,448 ± 4,251 mg/24 h; P = 0.29).

It was possible to evaluate treatment response in 118 cases (70.2%). Thirty-one patients (18.5%) was lost from the follow-up, and in 19 cases (11.3%), it was not possible to define a response after the end of the study.

**Figure 1.**
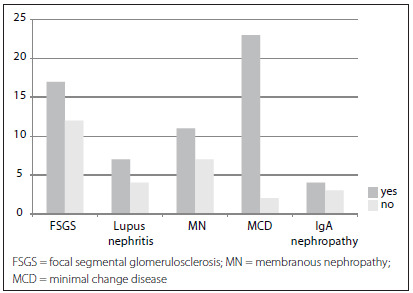
Response to treatment relating to the main glomerular diseases; P = 0.004, Fisher’s exact test.

The glomerular disease with the highest partial or complete remission rate was minimal change disease (92%). Figure 1 illustrates the partial and complete responses among glomerular diseases.

**Figure 2.**
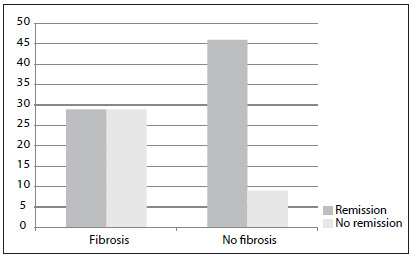
Relationship between interstitial fibrosis and treatment response among patients with glomerular diseases; P = 0.001, Fisher’s exact test.

Absence of interstitial fibrosis presented a strong correlation with remission: remission in patients without fibrosis = 83.4%, versus 16.3% in those with fibrosis; P = 0.001 (Figure 2).

## DISCUSSION

### Epidemiological characteristics

Epidemiological studies on glomerular diseases are rare in Brazil, but this subject is being debated with increasing frequency in our region. The present study evaluated patients who were followed up at a reference center in northeastern Brazil.

There was no difference in the prevalence of glomerular diseases between genders in the present study, and this is not in accordance with the findings from other studies in our country. Female gender was more prevalent in studies conducted in the northern Brazilian states of Amazonas and Pará (59% and 62.5%, respectively).^18^^,^^19^ In one study that included large numbers of patients from different Brazilian states (9,617), there was a slight predominance of females (51%).^20^

In the present study, the patients’ ages ranged from 14 to 77 years. The absence of younger patients in our cohort reflects the fact that children are followed up at another center, the pediatric hospital. The patients’ mean age was 37 years, which is in accordance with a previous study in Brazil.^6^

### Prevalence and classification of glomerular diseases

The most prevalent glomerular diseases were focal segmental glomerulosclerosis (19.6%), minimal change disease (17.9%) and membranous nephropathy (16.7%). Similar results were found in a study in the Amazon region, where focal segmental glomerulosclerosis was present in 33% of the cases, followed by membranous nephropathy, which was found in 20.9%.^18^ These data are also similar to the findings from other Brazilian studies.^20^

The prevalence of glomerular diseases presents high variability between different parts of the world.^3^^,^^4^^,^^5^^,^^6^^,^^14^^,^^19^^,^^20^^,^^21^^,^^22^^,^^23^ The majority of studies have found that idiopathic focal segmental glomerulosclerosis is the most common glomerular disease in adults.^6^^,^^20^^,^^24^ The reason for the variability in the prevalence of these diseases around the world remains unclear, but it is known that ethnic, socioeconomic and geographical factors may explain this.

The low prevalence of IgA nephropathy (7.1%) in the present study may be related to the fact that biopsies are not routinely performed on patients with hematuria alone.^20^ Patients with IgA nephropathy frequently present asymptomatic microscopic hematuria, and there is no need for specific therapy. The presence of proteinuria is the main factor associated with the need for therapeutic intervention in cases of IgA nephropathy, and this was the main indication for biopsy in our patients.^25^ IgA nephropathy is the most prevalent glomerular disease in some areas, such as Japan, Korea, China and France.^4^^,^^14^^,^^22^^,^^26^^,^^27^^,^^28^ In these countries, the investigations on glomerular diseases in patients with minimal urinary changes are more detailed, which would explain the high prevalence of IgA nephropathy found in some parts of the world.

Secondary glomerular diseases were more frequent in females (69%), which was probably due to the predominance of lupus nephritis in females. Investigation of infectious causes was positive in only two cases: one showing cured hepatitis B and one with HIV infection. In a previous study in China, the prevalence of glomerular disease in association with hepatitis B virus was also very low (1% of the cases), despite the high levels of hepatitis B virus infection in that country.^4^ HIV-associated nephropathy previously presented rapid evolution to nephrotic syndrome and renal failure, before the advent of antiretroviral therapy.^12^^,^^29^ After the introduction of protease inhibitors, the incidence of chronic kidney disease secondary to HIV/AIDS decreased.^29^

Lupus nephritis was the most frequent secondary glomerular disease, which was in accordance with previous studies.^3^^,^^4^^,^^14^^,^^19^^,^^22^^,^^30^ Among the patients with lupus nephritis, 18 (90%) were females, and their mean age was 34 years. The majority of the patients were between their third and fourth decades of life, which is similar to what has been described in the literature.^6^ The majority of the patients with lupus nephritis (60%) were categorized as class IV, which is also in accordance with previous studies.^6^^,^^12^

### Clinical manifestations

Similarly to reports in the literature, the main manifestation observed in the present study was nephrotic syndrome.^3^^,^^4^^,^^14^^,^^19^^,^^22^^,^^23^^,^^30^ Focal segmental glomerulosclerosis, minimal change disease and membranous nephropathy were the main diseases manifesting with nephrotic syndrome.

These findings are similar to those of previous studies. Morales et al., in a study on 157 patients in Porto Alegre, southern Brazil, found that focal segmental glomerulosclerosis and membranous nephropathy were the diseases that most frequently manifested with nephrotic syndrome.^31^ In the present study, focal segmental glomerulosclerosis was responsible for 22.1% of the cases with nephrotic syndrome, and this frequency can reach higher levels (up to 40%).^32^

IgA nephropathy was responsible for only 1.7% of the cases with nephrotic syndrome, which was lower than has been described in the literature. Previous studies found nephrotic syndrome in association with IgA nephropathy in 4.2 to 6.8%.^5^^,^^20^ One interesting finding in the present study was the fact that 58.3% of the patients with IgA nephropathy presented renal insufficiency on admission. This is not in accordance with previous studies, which described microscopic hematuria as the most common manifestation of this glomerular disease. This finding can be explained by the fact that our hospital is a reference center for nephrology, and this would account for the high number of patients with severe renal dysfunction.

Regarding renal function, we observed that 45.8% of the patients studied had low glomerular filtration rates on admission. These patients were of greater mean age than those with a normal glomerular filtration rate. The association between age and renal insufficiency was statistically significant, thereby showing that more advanced age is a risk factor for renal insufficiency among patients with glomerular diseases. There is a consensus that an increased creatinine level on admission may suggest a poor response to treatment in glomerular disease cases.^24^

In the present study, there was an improvement in glomerular filtration rate over the first six months after treatment began, and there was stabilization after one year of follow-up. This suggests that some patients had presented a low glomerular filtration rate on admission, due to acute kidney injury, probably caused by hypovolemia that was induced by the nephrotic syndrome.

There was no significant difference in the mean 24-hour proteinuria on admission between patients with and without renal insufficiency, thus showing that proteinuria is not associated with a decreased glomerular filtration rate.

### Prognostic factors

Patients with a good response to treatment had similar ages to those who did not respond (36.23 ± 13.59 versus 36.57 ± 15.21 years; P = 0.91), which showed that age is not a prognostic factor regarding the response to treatment in glomerular diseases.

Patients with a good therapeutic response presented similar proteinuria levels to those who did not respond (P = 0.29), and therefore proteinuria was not a predictor of poor prognosis in glomerular diseases. Previous studies have shown that the most important data for predicting the therapeutic response is not the initial levels of proteinuria, but the degree of reduction after treatment has begun.^33^

In the present study, absence of interstitial abnormalities in the renal biopsy was associated with a better prognosis. The majority of the patients who did not have any interstitial changes in their biopsy responded to treatment (83.6%), which is in accordance with previous studies.^31^ These studies showed that the factors associated with poor prognosis in glomerular diseases include initial resistance to corticosteroids, proteinuria levels, initial serum creatinine levels, glomerular sclerosis in more than 30% of glomeruli and the degree of interstitial fibrosis.^31^

### Study limitations

There are some limitations to the present study. The retrospective analysis of medical records was a limitation, since some data may have been missing. The small number of patients meant that it was not possible to perform a more powerful statistical analysis in order to assess the best treatment for the glomerular diseases studied. Nonetheless, the results presented here highlight some important aspects of the most prevalent glomerular diseases in our region and the pattern of treatment responses.

Renal biopsy remains the gold-standard method for diagnosing glomerular diseases and for guiding their treatment.^34^^,^^35^^,^^36^^,^^37^ In the majority of glomerular diseases, it is clinically impossible to predict the type or severity of the disease with good accuracy.

## CONCLUSIONS

The present study found that the most frequent glomerular diseases were focal segmental glomerulosclerosis, minimal change disease, membranous nephropathy and lupus nephritis. The presence of interstitial fibrosis was a predictor of poor therapeutic response. These results are important in that they provide better knowledge of glomerular disease patterns and guide future research on this subject. Biopsy findings suggestive of poor prognosis should increase medical attention to possible therapeutic unresponsiveness.
